# The Approach to Dental Caries Prevention in a Case of Agenesis of the Major Salivary Glands: A Case Report

**DOI:** 10.7759/cureus.52923

**Published:** 2024-01-25

**Authors:** Akram F Qutob

**Affiliations:** 1 Public Health and Pediatric Dentistry, King Abdulaziz University, Jeddah, SAU

**Keywords:** caries, dental, prevention, salivary, xerostomia

## Abstract

Xerostomia leads to great challenges for patients and dentists in managing and maintaining oral health due to the high risk of developing dental caries. We discuss a case of a 10-year-old male patient who presented with complaints of decayed teeth and difficulty chewing and swallowing food. He had bilateral congenital stenosis and stricture of lacrimal ducts and a family history of lacrimal gland agenesis and Hashimoto’s disease. The diagnosis reached was agenesis of all major salivary glands confirmed by saliva testing and ultrasound examination of the glands. Comprehensive preventative, restorative, and maintenance protocols based on caries management by risk assessment (CAMBRA) were implemented, including fissure sealants, amalgam and composite resin restorations, professional and home-applied fluoride, chlorhexidine mouthwash, frequent water consumption, and two-monthly recalls. We were able to stabilize the patient’s risk of dental caries for over three years. The implementation of stringent restorative, preventive, and maintenance protocols is key to improving and maintaining oral health in severe cases of xerostomia.

## Introduction

Xerostomia is defined as dryness of the mouth caused by decreased secretion of saliva. According to a systematic review involving individuals aged older than 18 years, the prevalence of xerostomia in this population ranges between 0.9% and 64% [1]. Many other studies have documented that the prevalence of xerostomia increases with age, certain medications, and other underlying diseases such as Sjögren's syndrome[2]. However, studies assessing the prevalence of xerostomia among children have been confined to those undergoing cancer treatment or any form of radiation-based therapy or those diagnosed with diseases that impair the function of the salivary glands [3].

The causes of xerostomia can be classified into salivary and non-salivary [4]. The non-salivary causes include dehydration, cognitive alteration, neurological or oral sensory dysfunction, psychological issues, and mouth breathing due to obstruction in the upper airway tract. On the other hand, salivary causes include diseases such as autoimmune disorders (e.g., Sjögren's syndrome, systemic lupus, sarcoidosis, rheumatoid arthritis, scleroderma, and primary biliary cirrhosis), diabetes mellitus, HIV, herpes virus, hepatitis C, and end-stage renal disease. Other salivary causes include congenital and hereditary factors, treatment side effects, salivary gland trauma or tumor, and nutritional deficiencies/eating disorders. This case was diagnosed under the category of congenital causes, which may include either hypoplasia or aplasia of salivary glands. Aplasia of salivary glands is associated with either hypoplasia or aplasia of lacrimal glands [5-7]. These patients can experience multiple side effects including an increased risk of dental caries, oral candidiasis, ascending sialadenitis, laryngitis, pharyngitis, and difficulty in swallowing [8].

## Case presentation

The patient was a 10-year-old male referred by a local dentist for assessment and management of dry mouth and multiple carious teeth. His past medical history revealed bilateral congenital stenosis and stricture of lacrimal gland ducts in addition to agenesis of superior lacrimal canaliculi. The patient suffered from dry eyes, seasonal asthma, and bilateral mild fixed flexion knee deformities. His medications included hydrating eye drops and occasional use of Ventolin. His family history showed that several family members from the father's side suffered from agenesis of lacrimal glands, suggesting a genetic component. On the patient’s mother's side, his great-grandmother had been diagnosed with Hashimoto’s disease, characterized by fatigue, a poor ability to tolerate cold, joint/muscle pain, and dry skin and hair. His mother had multiple symptoms suggestive of autoimmune conditions including Hashimoto’s disease, rash, photosensitivity, and arthritis with a possible unifying diagnosis of systemic lupus erythematosus.

The patient's past dental history included regular dental care visits at a local dentist since the age of five years. During this period, the patient had multiple glass ionomer restorations, pulpotomy treatments, and extractions for multiple carious primary teeth. His first permanent molars had become carious soon after the eruption and had been treated by resin-based fissure sealant. He had also received multiple oral hygiene-related instructions, diet counseling, and professionally applied topical fluoride at the local dentist. His dry mouth had been initially noticed two years before his referral to the pediatric dentistry consultant. Since the patient’s referral, he had been following regular oral hygiene practices of brushing with fluoridated toothpaste twice a day. His diet content and frequency of snacking did not seem to be cariogenic, with adequate breaks between meals and snacks.

**Figure 1:**
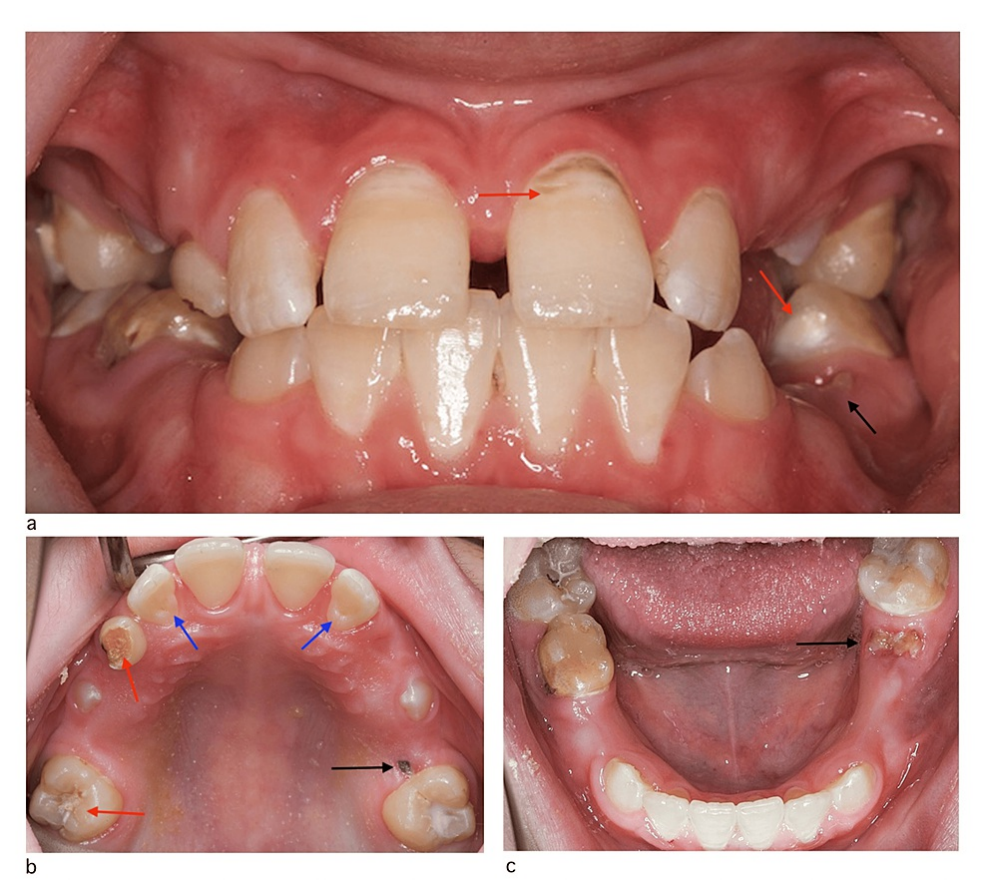
Intraoral clinical photos before treatment a) Intraoral clinical photo frontal view. b) Intraoral clinical photo of the maxillary arch. c) Intraoral clinical photo of the mandibular arch Red arrows: demineralized enamel lesions and cavitated dental caries. Black arrows: remaining roots. Blue arrows: deep palatal pits

Extra and intraoral examinations were performed and the patient’s oral hygiene was rated as fair with an absence of gingival inflammation and dental plaque. However, there was a reduction in the patient’s saliva production, evidenced by overall dryness of the oral mucosa and slow saliva secretions after drying the lower lip. The patient was in his late mixed dentition stage of dental development with class I molar relationship. He had partially erupted teeth (#14 and #24) and deep palatal fissures on teeth (#12 and #22). He also had multiple carious primary teeth, primary teeth root stumps, and demineralization on the mesial surface of tooth #36 and the cervical surfaces of most of his permanent teeth. Cavitated clinical caries was evident on the mesial and distal surfaces of tooth #31 and the mesial surface of tooth #41. Existing restorations included defective resin-based fissure sealants on the first permanent molars and large occlusal composite resin restoration on tooth #85 (Figure 1).

**Figure 2:**
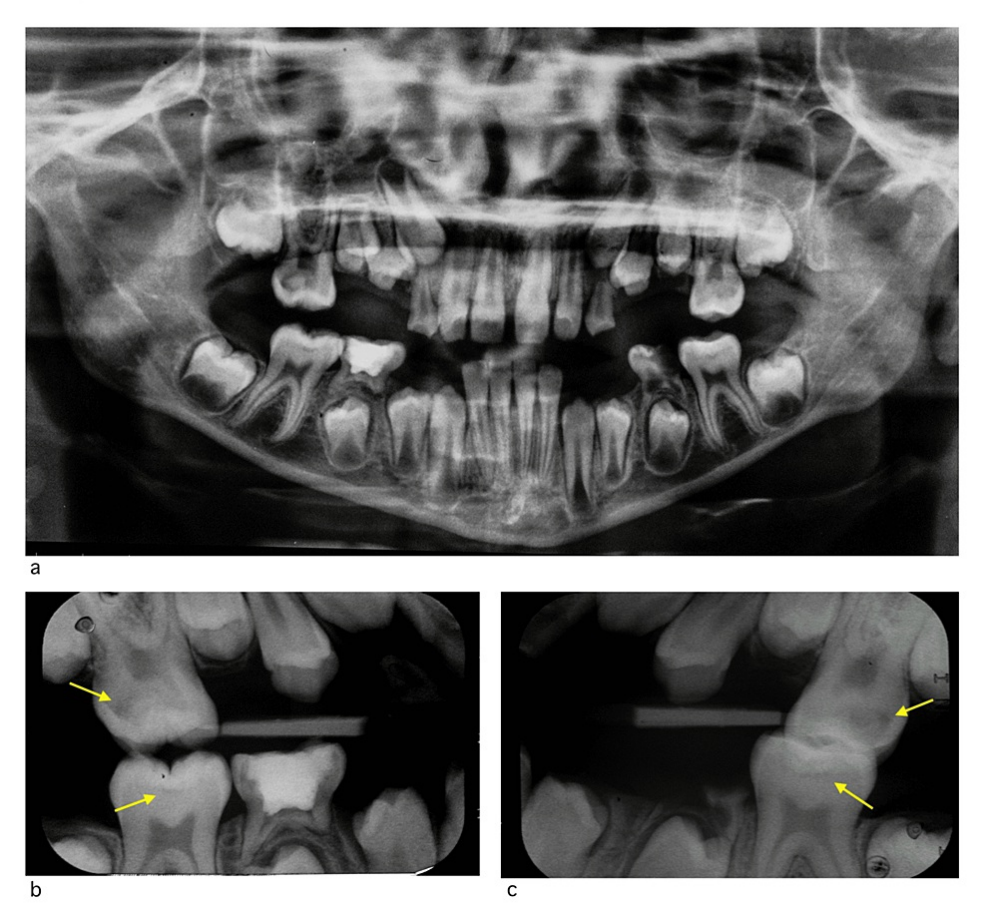
OPG and bitewing radiographs before treatment a) OPG radiograph. b) Right-side bitewing radiograph. c) Left-side bitewing radiograph Yellow arrows: recurrent dental caries under existing restorations/fissure sealants OPG: orthopantomogram

Radiographic examination including orthopantomogram (OPG) and bitewing radiographs were obtained and showed normal dental development, absence of evidence of salivary gland stones, multiple deep and initial carious lesions in primary and permanent teeth, and remaining roots (Figure 2).

**Figure 3:**
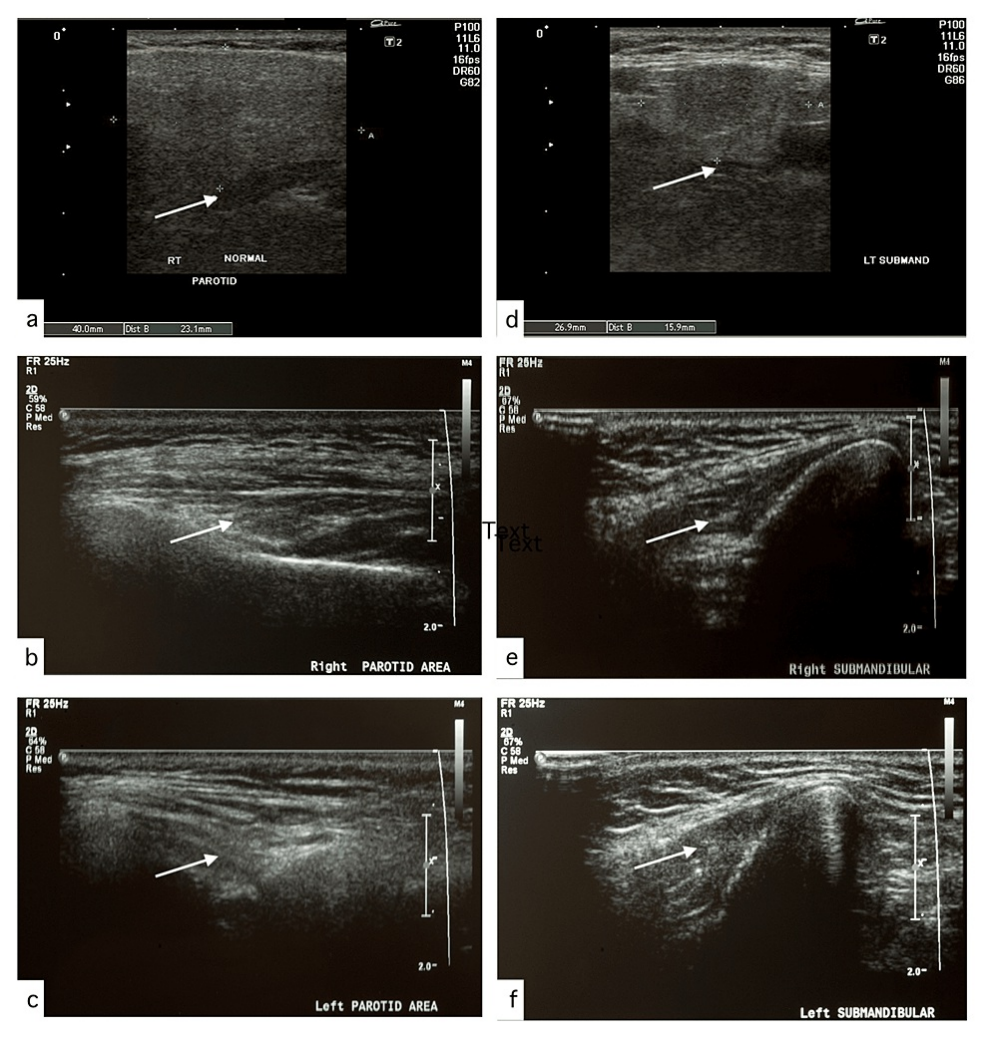
Ultrasonography of normal and missing right and left parotid and submandibular salivary glands a) Ultrasonography of normal parotid gland. b) Ultrasonography of missing right parotid gland. c) Ultrasonography of missing left parotid gland. d) Ultrasonography of normal submandibular gland. e) Ultrasonography of missing right submandibular gland. f) Ultrasonography of missing left submandibular gland White arrows: location of salivary glands with distinction between normal images (a and d) and images with missing salivary glands (b, c, e, and f)

Saliva testing was performed multiple times by using a GC saliva-check buffer kit to determine the various aspects of the quantity and quality of saliva. Resting saliva was examined through visual inspection of the level of hydration, which was low, indicating a low resting flow of saliva. Saliva consistency was frothy and bubbly, indicating an increased viscosity of saliva. The quantity of stimulated saliva at five minutes was <3.5 ml, indicating a very low stimulated saliva flow. The buffering capacity of saliva was found to be very low (scores between 0-5 as compared to a normal score of 10-12), and the saliva pH measurement was 6.2, indicating moderately acidic saliva. Special imaging for salivary glands was performed including ultrasonography, which revealed bilateral absence of all major salivary glands (parotid and submandibular) (Figure 3).

**Figure 4:**
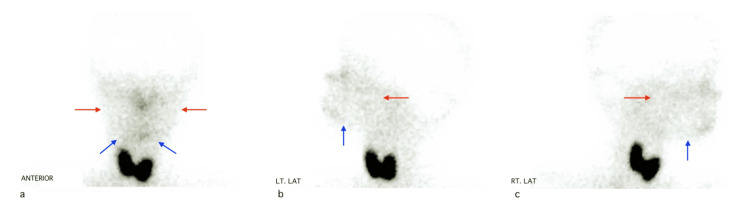
Nuclear medicine scan of major salivary glands showing no uptake a) Anterior view showing no uptake by major salivary glands. b) Left view showing no uptake by major salivary glands. c) Right view showing no uptake by major salivary glands Red arrows: position of parotid salivary glands. Blue arrows: position of submandibular salivary glands

**Figure 5:**
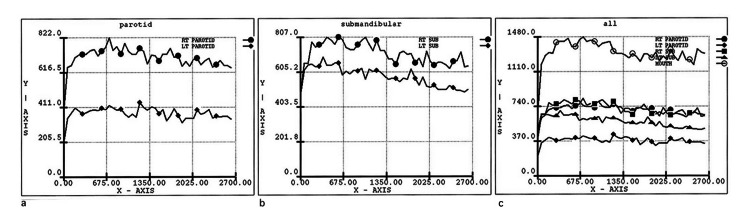
Major salivary glands: function (secretion and uptake) graph a) Salivary gland function graph of the parotid gland. b) Salivary gland function graph of the submandibular gland. c) Salivary gland function graph of the whole mouth

A nuclear medicine scan was undertaken at King Abdulaziz University Hospital in Jeddah to test major salivary glands’ function. The results confirmed a bilateral absence of secretion and uptake of parotid and submandibular salivary glands (Figures 4, 5).

Medical consultation with rheumatology did not show any evidence of autoimmune disease. However, the patient was scheduled for regular reviews for possible late presentation of an autoimmune disease. He was also referred to medical genetics to investigate the possibility of a hereditary cause of xerostomia, e.g., aplasia of the lacrimal and major salivary glands (ALSG), and the results came back negative for any genetic component related to his condition.

Based on previous clinical and imaging findings, the patient was diagnosed with complete bilateral agenesis of all major salivary glands (parotid, submandibular, and sublingual) as evident on ultrasonography. A comprehensive treatment plan was drawn up and involved prevention, restorations/remineralization, and regular follow-ups. Caries management by risk assessment (CAMBRA) protocol was used throughout the patient’s dental management plan due to its strong scientific evidence and comprehensiveness in its recommendations [9]. Prevention measures focused on improving brushing skills to remove dental plaque twice a day, using high-fluoride concentration toothpaste [5000 part per million (ppm)], maintaining low bacterial counts by using 0.2% chlorhexidine mouthwash (used once a day for one week and repeated every month) and sipping water frequently to keep the mouth hydrated and using saliva stimulants, e.g., sugar-free gum. Fissure sealants were placed on palatal fissures of teeth #12 and #22, as well as any newly erupting teeth.

**Figure 6:**
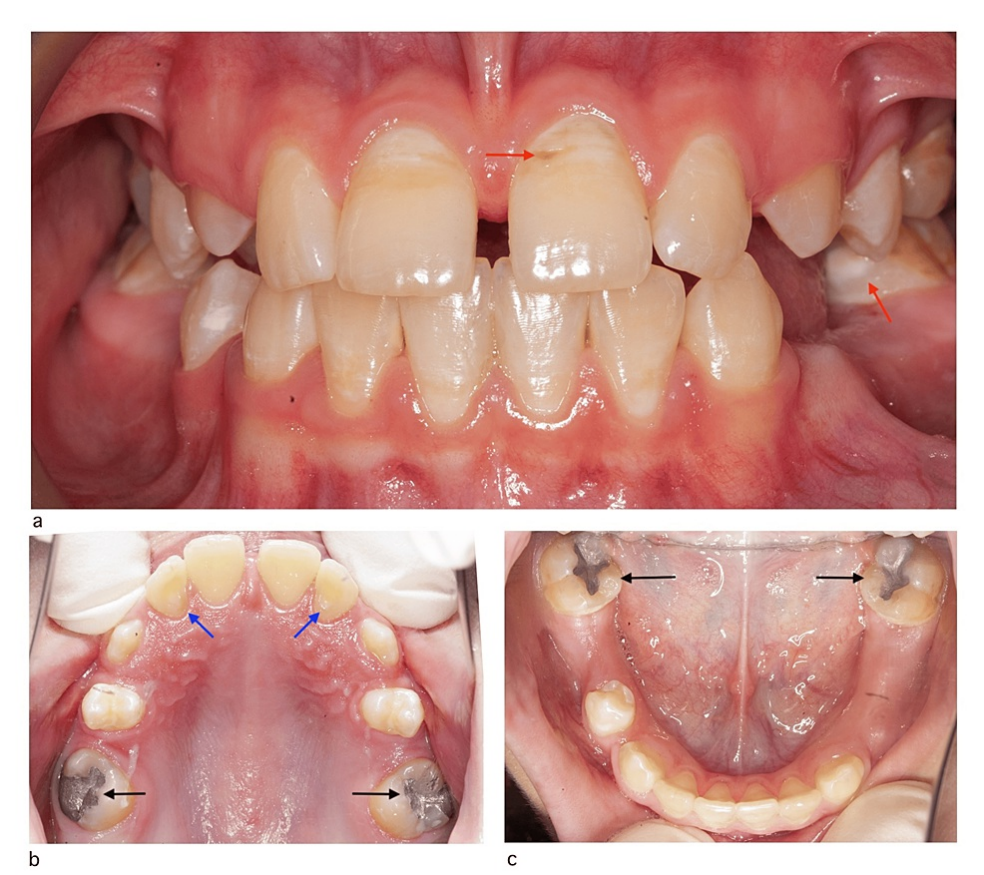
Intraoral clinical photos after treatment a) Intraoral clinical photo frontal view. b) Intraoral clinical photo of the maxillary arch. c) Intraoral clinical photo of the mandibular arch Black arrows: amalgam restorations. Blue arrows: sealants on palatal fissures. Red arrows: non-cavitated demineralized enamel lesions

**Figure 7:**
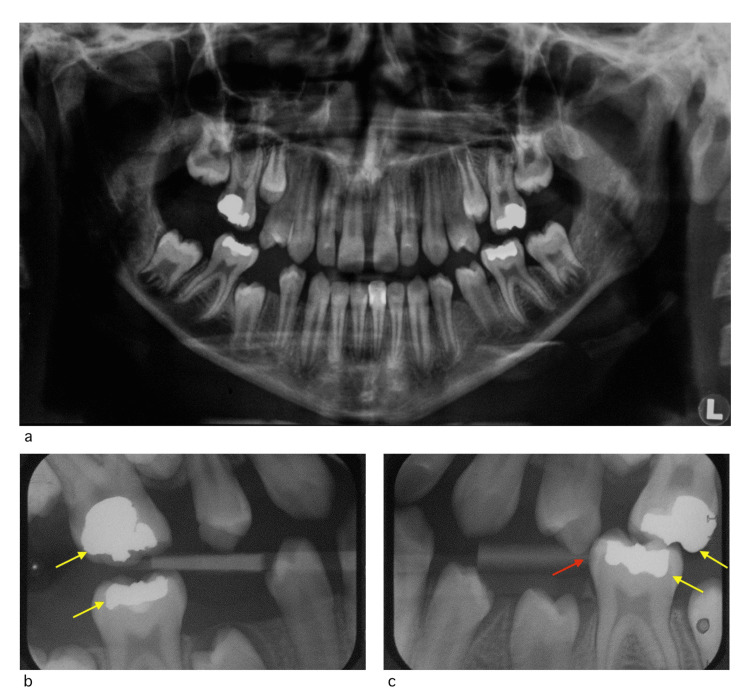
OPG and bitewing radiographs after treatment a) OPG radiograph. b) Right-side bitewing radiograph. c) Left-side bitewing radiograph Yellow arrows: amalgam restorations. Red arrows: demineralized non-cavitated enamel lesions

Restorative treatments involved extracting carious primary teeth and remaining roots at the outset. This was followed by placing amalgam restorations on permanent molars and composite resin restorations on anterior teeth. Remineralization of non-cavitated carious lesions was achieved by daily home application of high-fluoride concentration toothpaste (5000 ppm) in special trays to be applied one hour before bedtime and by monthly application of fluoride varnish on all teeth. At the end of the remineralization therapy, the patient was placed on a regular follow-up and maintenance plan every three months, which involved caries monitoring and reinforcing home and professional preventive care. The follow-up was conducted for three consecutive years during which no new carious lesions developed, and oral hygiene was stabilized (Figures 6, 7).

## Discussion

The correct diagnosis of xerostomia depends on accurate history taking, physical examination, testing of saliva quantity and quality, medical consultation to rule out underlying autoimmune diseases, salivary gland imaging, and, at last, biopsy. This case was considered a rare one, as described earlier in the introduction section. The steps taken to diagnose the condition ranged from simple observation of the presence of saliva in the mouth, followed by saliva testing, to more advanced investigations, e.g., ultrasonography, nuclear medicine scan, and referral to medical genetics. This gradual and logical method of investigation helped reduce patient discomfort and cost, and at the same time reduced the use of more advanced and expensive hospital resources.

The comprehensive dental treatment and management plan using sound scientific evidence was crucial in the success of maintaining good oral health for this compromised patient. Adopting the CAMBRA protocol had a great impact on the overall success of this case over three years. Amalgam restorations are less frequently used nowadays due to patient preferences and advancements in composite resin materials and indirect restorative options, e.g., porcelain inlays/onlays. However, as compared to composite resin restorations, the properties, longevity, success rates, and lower cost of amalgam restorations make them a superior choice in certain cases of extreme xerostomia [10,11]. An argument can be made for the choice of using mostly composite resin restorations instead of glass ionomer restorations in restoring the interproximal lesions of the mandibular anterior teeth. Although the patient may benefit from the fluoride-releasing characteristic of glass ionomer restorations, the preventive regimen that we adopted included frequent application of high-concentration fluoride agents and was deemed superior to the minimal fluoride release from the glass ionomer restorations, thereby justifying the use of composite resins.

In this case, the preventative regimen focused on restoring the imbalance caused by the lack of salivary secretion that had placed the patient in the extreme risk category for developing dental caries. The preventive regimen included cariostatic agents (0.2% chlorhexidine mouthwash), remineralizing agents (high-fluoride concentration toothpaste and professional application of fluoride varnish), diet counseling with oral hygiene instructions, use of saliva stimulants, and frequent dental visits. The patient and his parents were highly enthusiastic about the preventive and remineralization therapies. After three years of follow-up, the patient did not develop new carious lesions. The size of the mesial lesion on tooth #32 remained the same and there was evidence of remineralization of the other demineralizing lesions (mesial surfaces of teeth #36 and #46). The fillings were all intact except for marginal staining around the margins of the composite restoration of tooth #31, which was replaced by a composite resin strip crown.

Reinforcing oral hygiene, home care, and the family's willingness to follow the dentist's instructions and advice had a remarkable impact on the overall success of this case. It is also worth mentioning that taking into account the social behavioral background of the patient in the formulation of the treatment plan, choice of restorative materials, and the simplification of oral home care played a huge role in ensuring patient compliance throughout the treatment course and maintenance journey.

## Conclusions

Following a well-structured and evidence-based dental management is essential to maintain good oral health in patients suffering from severe xerostomia. Such cases of agenesis of all major salivary glands are extremely rare, and it takes great efforts from the dentist and the patient/patient’s family to prevent dental caries in these high-risk patients. Existing evidence-based protocols such as CAMBRA have been found to be successful in maintaining oral health and preventing dental diseases in such cases. After all, dentists should be skilled in finding the cause of dental caries in their patients and should not just rely on traditional oral hygiene instructions without investigating the root cause of the illness.
